# Emerging Approaches to Investigate the Influence of Transition Metals in the Proteinopathies

**DOI:** 10.3390/cells8101231

**Published:** 2019-10-10

**Authors:** Frederik Lermyte, James Everett, Jake Brooks, Francesca Bellingeri, Kharmen Billimoria, Peter J. Sadler, Peter B. O’Connor, Neil D. Telling, Joanna F. Collingwood

**Affiliations:** 1 School of Engineering, University of Warwick, Coventry CV4 7AL, UK; f.lermyte@warwick.ac.uk (F.L.); j.everett@keele.ac.uk (J.E.); j.brooks.1@warwick.ac.uk (J.B.); k.billimoria@warwick.ac.uk (K.B.); 2 Department of Chemistry, University of Warwick, Coventry CV4 7AL, UK; f.bellingeri@warwick.ac.uk (F.B.); p.j.sadler@warwick.ac.uk (P.J.S.); p.oconnor@warwick.ac.uk (P.B.O.); 3 School of Pharmacy and Bioengineering, Keele University, Stoke-on-Trent, Staffordshire ST4 7QB, UK; n.d.telling@keele.ac.uk; 4 MAS-CDT, University of Warwick, Coventry CV4 7AL, UK; 5 Materials Science and Engineering, University of Florida, Gainesville, FL 32611, USA

**Keywords:** Alzheimer’s disease, Parkinson’s disease, amyloid β, α-synuclein, copper, iron, mass spectrometry, electrospray ionization, X-ray, spectromicroscopy

## Abstract

Transition metals have essential roles in brain structure and function, and are associated with pathological processes in neurodegenerative disorders classed as proteinopathies. Synchrotron X-ray techniques, coupled with ultrahigh-resolution mass spectrometry, have been applied to study iron and copper interactions with amyloid β (1–42) or α-synuclein. Ex vivo tissue and in vitro systems were investigated, showing the capability to identify metal oxidation states, probe local chemical environments, and localize metal-peptide binding sites. Synchrotron experiments showed that the chemical reduction of ferric (Fe^3+^) iron and cupric (Cu^2+^) copper can occur in vitro after incubating each metal in the presence of Aβ for one week, and to a lesser extent for ferric iron incubated with α-syn. Nanoscale chemical speciation mapping of Aβ-Fe complexes revealed a spatial heterogeneity in chemical reduction of iron within individual aggregates. Mass spectrometry allowed the determination of the highest-affinity binding region in all four metal-biomolecule complexes. Iron and copper were coordinated by the same N-terminal region of Aβ, likely through histidine residues. Fe^3+^ bound to a C-terminal region of α-syn, rich in aspartic and glutamic acid residues, and Cu^2+^ to the N-terminal region of α-syn. Elucidating the biochemistry of these metal-biomolecule complexes and identifying drivers of chemical reduction processes for which there is evidence ex-vivo, are critical to the advanced understanding of disease aetiology.

## 1. Introduction

There is a long-established association between aberrant protein deposition in neurodegenerative disorders, and disrupted metabolism of metals. The extent to which metal-protein interactions are a contributing factor in disease pathogenesis remains an active research question, arguably due to the complexity of the systems concerned and the level of analytical challenge associated with their study. 

The proteinopathies include many forms of dementia and movement disorders, and disease-specific patterns of aberrant protein deposition are integral diagnostic hallmarks in disorders such as Alzheimer’s disease, Parkinson’s disease, dementia with Lewy bodies, multiple system atrophy, and others. A causative role has been postulated for the well-documented protein cleavage and misfolding that arises in disorders, such as Alzheimer’s and Parkinson’s disease (for example, the amyloid cascade hypothesis [1]), where increased central nervous system (CNS) concentrations and subsequent aggregation of amyloid β, a 4.5 kDa peptide, and α-synuclein, a 14 kDa protein, are observed. The hallmarks of these diseases include amyloid β forming extracellular deposits as senile plaques, and α-synuclein intracellular aggregates (Lewy bodies), respectively. The patterns and forms of amyloid and synuclein deposition are a factor in post-mortem classification of these neurodegenerative disorders. Attempts to intervene in the disease progression by targeting amyloid burden in isolation (typically using selective monoclonal antibodies) have consistently failed [2,3,4,5]. 

Metals play many essential roles in the human brain [6,7]. There is long-standing, but incomplete, evidence of disrupted metal ion metabolism and localized accumulation in neurodegenerative disorders (including the abundant biometals such as calcium, the essential transition metals, and non-essential metals that can enter the CNS such as aluminium). These observations have motivated the development of chelating drugs. Clinical trials of approaches successful in treating systemic overload disorders (e.g., iron chelation in haemochromatosis, copper chelation in Wilson’s disease), have been extended to disorders primarily of the CNS. Chelation trials to date have shown mixed results for the application to neurodegenerative disorders, but some have delivered promising results in modulating iron and copper [8,9,10]. 

In this context, it is of fundamental importance to consider the interplay between metals and peptides. It is well-established that amyloid β and α-synuclein deposits in human brain tissue are associated with metal accumulations, and these metals can affect the aggregation kinetics of amyloidogenic peptides and proteins through the induction of conformational change and/or metal-catalysed oxidation of the protein backbone [11,12,13,14,15,16,17,18,19,20,21,22,23]. It has been postulated that binding of metallic counter-ions neutralises charge repulsion, permitting the formation of more compact and structured conformations, such as those that comprise filamentous Lewy bodies [23]. In vitro studies have demonstrated accelerated α-synuclein fibrillation associated with both iron [11,24] and copper [11,23,25] co-incubation.

Furthermore, it has been shown in ex vivo tissue studies that the metal-containing phases within senile plaques and Lewy bodies are often chemically reduced and therefore reactive, suggesting that the metal-catalysed production of radicals and reactive oxygen species might contribute to neuron death [13,14,20,26,27,28,29,30]. There is, therefore, a pressing need to understand the interactions of transition metal ions with amyloid β and α-synuclein on a molecular scale, including the way these interactions affect the oxidation states of the metals, supported by the evidence of chemical reduction in-vitro or within cultured cells [26,31,32].

**Figure 1:**
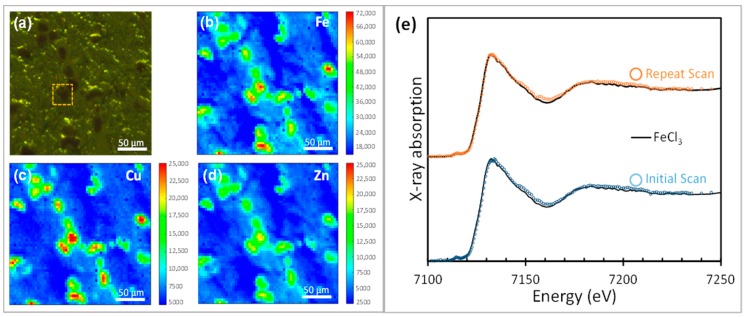
(**a**) Optical image showing melanised dopaminergic neurons in PD substantia nigra tissue. X-ray fluorescence maps of the area shown in (**a**) were collected using a 5 µm beam and a 0.1 mm Al foil attenuator. XRF maps are shown for (**b**) iron, (**c**) copper, and (**d**) zinc at their respective K-edges. (**e**) Iron XANES from the individual neuron highlighted in (**a**), showing successive scans on the same area to monitor possible photo-reduction, with the results from linear combination fitting of XANES spectra alongside experimental standards (a range of Fe^0^/Fe^2+^/Fe^3+^ standards and ferritin-bound Fe^3+^ iron). The repeated traces were identical within measurement uncertainty (<2%), and the absorption edge region (−20 eV below to +80 eV above the edge) fitted well with 100% FeCl_3_, with χ^2^ values of 0.11 and 0.07 obtained for the initial and repeat scans, respectively. Traces were subjected to an edge-step normalization and flattened using Athena fitting software. Traces are vertically offset for clarity.

Studying the spatial distribution of trace metals in solid samples (such as brain tissues and their derivatives) poses significant challenges, and only a handful of methods can simultaneously provide information on the metals’ chemical state. Synchrotron X-ray methods have received considerable attention in this context [7,33]. X-ray techniques using hard, and more recently soft, X-rays have been successfully utilized to examine neurodegenerative disease tissues ex vivo [7,13,34,35,36]. Utilizing unique structures and natural contrast in samples is important to maximise the success of these studies where non-destructive methods are employed, as the same region of a sample may be analysed multiple times. In the example of brain tissue analysed for this article (Figure 1), neurons from the substantia nigra, naturally pigmented with granules of neuromelanin (NM), were used, as the metabolism of metals in this region is of interest, particularly in the context of Parkinson’s disease [37,38,39] and NM—a biological polymer formed by autoxidation of dopamine—provides useful contrast. 

Micro-focus X-ray fluorescence (μXRF) mapping provides a means to establish simultaneously the distribution of multiple trace metal elements at spatial resolutions of a few microns. Synchrotron X-rays can also be tuned in energy with great precision to excite electrons from core atomic shells, causing an abrupt increase in photo-absorption at energies equal to the shell binding energy [7,40]. This increase is recognized as an ionization edge in the X-ray absorption spectrum, and an analysis of the characteristic shape of the absorption spectrum near the ionization edge is known as X-ray absorption near-edge spectroscopy (XANES). XANES focuses on the absorption fine structure near the edge corresponding to the excitation energy of an inner-shell electron to an unoccupied orbital. When probing metal *K*-shell electrons, this typically requires the use of a high-energy (> 5 keV/photon) hard X-ray beam, while metal *L*-shell electrons can be probed using a lower energy soft X-ray beam (< 5 keV). These X-ray techniques have the benefit of providing information on the metal oxidation state, and are sensitive to the types and numbers of coordinated ligand atoms and metal-ligand distances. With sufficiently pure samples, it may be possible to determine the structures from first principles using extended X-ray absorption fine structure (EXAFS). XANES analysis with a micro-focused beam may be performed in situ without the requirement for histological staining or contrast agents (which are likely to alter the native biochemistry), and for the equivalent spatial resolution, the typical beam damage is lower from synchrotron X-ray methods than from conventional electron beam methods [7]. XANES spectra can be collected in the transmission or fluorescence mode, though the fluorescence mode is typically preferred for low (approximately ppm) concentrations [40], and XANES acquisition can be informed by prior XRF mapping, or even integrated into a XANES mapping experiment at many micro-focus beamlines.

A further synchrotron X-ray approach emerging as a powerful tool for the analysis of heterogeneous biological samples is soft X-ray spectromicroscopy, in the form of scanning transmission X-ray microscopy (STXM). This approach combines spectroscopic analysis with high-resolution microscopy enabling the chemical speciation of a sample material to be determined to a spatial resolution of approximately 20 nm. The operational energy range of STXM microscopes spans both organic (e.g., C, together with N, O and P) and inorganic (metal) absorption edges, allowing for the correlations between metal chemistry and specific biological structures to be identified. Furthermore, magnetically sensitive STXM X-ray magnetic circular dichroism (XMCD) allows for site-specific magnetic characterization of metal phases within imaged regions of interest [13]. 

These X-ray methods are often considered non-destructive as, unlike imaging mass spectrometry methods, they do not rely on ablation of the sample. Sample preservation facilitates correlative imaging (for example, light and electron microscopy) to be performed following X-ray analysis. In addition, these approaches require no aldehyde fixatives, contrast agents or dyes, thereby preserving native sample chemistry to a greater extent than traditional approaches used to image biological tissues [41]. However, despite their non-destructive nature, it is necessary to carefully control the X-ray dose used in this type of experiment to ensure a reliable and reproducible chemical analysis. 

Mass spectrometry (MS) is an important technique used to study metalloproteins on a molecular (atomic) scale, particularly in combination with electrospray ionisation (ESI) [42,43,44,45,46]. Under optimised conditions, ESI can transfer intact metal-protein complexes into the gas phase, where their molecular mass can be measured very accurately. This also allows the determination of the oxidation state of the metal. For a metal-free protein *M*, the electrospray process produces a multiply charged cation *[M + nH]^n+^*. In other words, the charges are provided by the addition of protons, each possessing a mass of approximately 1.0073 Da. If a multiply-charged metal ion is attached to the protein, the ion produced instead is *[M + Metal + nH]^m+^*, and the oxidation state of the metal equals (*m*−*n*), i.e., the charges not accounted for by the proton addition are carried by the metal, the mass of which is essentially independent of the oxidation state. While this approach is very straightforward, two caveats should be noted. First of all, the ESI source is essentially an electrochemical cell, and redox reactions within this cell cannot be ruled out a priori. Second, as ionisation typically occurs under atmospheric conditions, there is a window of a few milliseconds during which the oxidation of very sensitive species can occur. Furthermore, ESI-MS requires highly purified samples in solution, and is incompatible with most of the non-volatile buffers and salts commonly used in molecular biology. Despite these limitations, ESI-MS offers several unique benefits, as different co-occurring protein isoforms and metal-bound states can be resolved, and these can be isolated for further analysis in tandem MS experiments. In this approach, an ion is isolated and subjected to gas-phase activation, for which several methods exist [47,48,49]. This induces the fragmentation of the amide backbone of the peptide or protein, and the masses of the resulting fragments are measured. By carefully controlling the fragmentation, it is possible in some cases to cleave the backbone while preserving protein-metal interactions, and as a result, the fragments that contain the residue(s) that interact(s) with the metal, show a characteristic mass increase [50,51,52,53]. As such, careful analysis of the fragmentation pattern can provide clues about the sequence region in which binding occurs.

This work demonstrates a suite of complementary approaches (Table 1) including X-ray spectromicroscopy, mass spectrometry, and supporting electron microscopy, to investigate the properties of metals interacting with peptides integral to the hallmarks of neurodegenerative disorders. 

In the example applications, unstained (label-free) post-mortem brain tissue was pre-imaged using μXRF to determine the oxidation state of iron in the regions of interest. Subsequently, hard X-ray XANES and soft X-ray spectromicroscopy, ESI-MS, and electron microscopy were used to analyse complexes of iron and copper with Aβ(1–42) and α-syn formed in vitro. In particular, the chemical environment of the metals in these complexes (i.e., the primary amino acid residue binding sites) was studied as well as the effect of binding and co-incubation on the oxidation state of the metals. A potential concern when using energetic X-ray probes to measure oxidation state chemistry is the possibility of X-ray photo-reduction. There are a few systematic reports into X-ray beam induced changes to sample chemistry in these systems of interest, but this concern is critical if robust insights are to be obtained. This study showed through a careful analysis and control of the X-ray exposure conditions, how such effects can be identified and successfully mitigated against.

## 2. Materials and Methods

### 2.1. Materials for In-Vitro Experiments

Synthetic Aβ(1–42) was acquired from Bachem (Bubendorf, Switzerland), the same source the authors have used in previous research [28,29]. The peptide was produced by solid-phase peptide synthesis using the Fmoc strategy followed by trifluoroacetic acid-mediated cleavage. Recombinant 140-residue α-synuclein (catalogue #AG938), CuCl, CuSO_4_, FeCl_2_, FeCl_3_, Fe(III) ammonium citrate, glutathione, 1,10-phenanthroline, nitrilotriacetic acid, and hydroxylamine hydrochloride were purchased from Sigma (Dorset, UK). As described in Section 3.3, one of the key techniques used in this work was ultrahigh-resolution tandem mass spectrometry, enabling the measurement of the masses of the intact Aβ(1–42) and α-syn as well as fragments produced in the gas phase with accuracy better than 1 part-per-million (see Table A1, Table A2, Table A3 and Table A4 in Appendix D). Hence, these data establish the high purity as well as the mass and sequence of the α-syn used in this work. 

Peptide and metal stocks were diluted in a modified Krebs-Henseleit (KH) buffer (pH 7.4; 100 mM PIPES, 118.5 mM NaCl, 4.8 mM KCl, 1.2 mM MgSO_4_, 1.4 mM CaCl_2_, 11 mM glucose and all Sigma Aldrich) modelled on the abundance of these elements reported in the cerebrospinal fluid of the central nervous system [17]. PIPES was utilized as a buffering agent as it does not interact strongly with metal ions.

#### 2.1.1. For XANES Experiments

**Figure A1:**
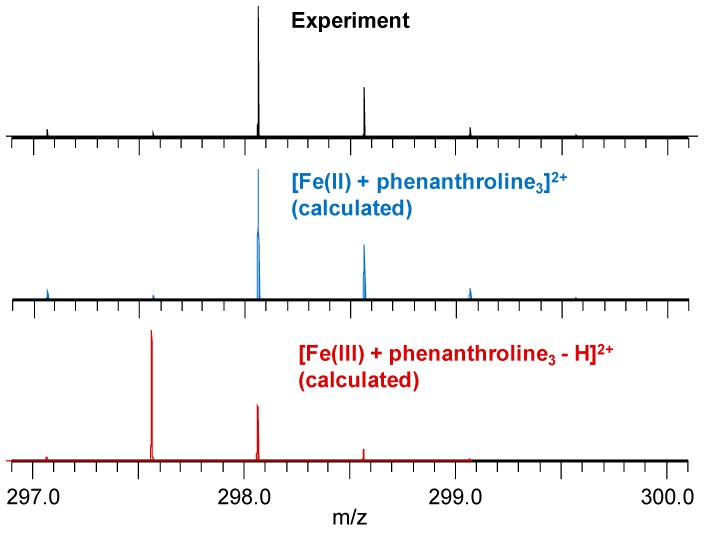
Mass spectrometry of [Fe(II) + 1,10-phenantroline_3_]^2+^ (10 µM in H_2_O with 20 mM ammonium acetate) demonstrates stabilization of the Fe(II) oxidation state under these conditions.

As the Fe(II) ion in aqueous solution is sensitive to oxidation by atmospheric oxygen, a stable Fe(II) reference standard for XANES was produced by making a solution containing a 1:3 molar ratio of FeCl_2_:1,10-phenanthroline, as, in the resulting complex, this ligand protects the iron center from oxidation. The Fe(II) oxidation state of iron in this complex was confirmed using MS (Appendix A Figure A1). Similarly, to stabilize the Cu(I) ion in aqueous solution, the appropriate amount of anhydrous CuCl to yield a concentration of 0.77 mM was dissolved in a 3.25 mM solution of glutathione (i.e., resulting in a 1:4 mol ratio), following a published procedure [54]. 

#### 2.1.2. For MS Experiments 

The reducing agent hydroxylamine hydrochloride was added in a 1:2 ratio with the metal to stabilize Fe(II) on the required timescale. To stabilize Fe(III) in solution at pH 7.4, a 1:1 complex with nitrilotriacetate (NTA) was used [55]. This complex was produced by dissolving FeCl_3_ and H_3_NTA—both at a concentration of 10 mM—in water, and subsequently adjusting the pH to 7.4 by the dropwise addition of 1 M aqueous ammonia.

#### 2.1.3. For STXM Experiments

Frozen synthetic Aβ(1–42) was thawed and dissolved in 0.1 M NaOH for 30 min at room temperature to create a 1 mg/mL (220 µM) Aβ stock. NaOH was used to dissolve Aβ aggregates (that would otherwise be insoluble at pH 7) that may have formed during the sample storage, ensuring complete peptide dissolution. Amyloid β stock was diluted in modified KH buffer (pH 7.4) and was allowed to incubate at 37 °C for 48 h before the addition of 18 mM iron(III) nitrate solution. Following the addition of iron(III), the suspensions of Aβ/iron were incubated at 37 °C for a further 30 min before sampling. The final Aβ and iron concentrations were 35 and 370 µM, respectively. The suspensions of Aβ/iron were incubated within sealed microcentrifuge tubes.

### 2.2. Human Tissue

Human brain tissue from the substantia nigra of a confirmed case of Parkinson’s disease was obtained fresh-frozen from the Canadian Brain Tissue Bank and cryosectioned with an acid-washed sapphire blade (to avoid any metal contamination) to a thickness of 30 μm, mounted on a spectroscopically-clean synthetic quartz slide, and air-dried in a class II hood prior to hard X-ray XRF and XANES analysis. The use of this tissue was conducted in accordance with the Declaration of Helsinki, under approval 07.MRE08.12 from North West Haydock Park Research Ethics Committee.

### 2.3. Hard X-ray Spectromicroscopy

Hard X-ray synchrotron XANES data, preceded by μXRF data, where required for the localization of regions of interest, were collected on the microfocus beamline I18 at the Diamond Light Source (Oxfordshire, UK). 

#### 2.3.1. For μXRF

A primary incident synchrotron X-ray beam of 13 keV was used to stimulate fluorescence emission simultaneously from all accessible elements in each sample analyzed, with the full fluorescence spectrum acquired for each pixel of every map. A focused X-ray beam diameter of 5 μm^2^ defined the maximum spatial resolution used. An optical camera was focused on the sample during mapping to allow the metal distribution to be correlated with anatomical tissue structure. All fluorescence maps were generated after using PyMCA software to subtract the background and fit the individual peaks. 

#### 2.3.2. For XANES

A series of solutions was prepared for analysis containing 35 µM Aβ or α-syn, and 440 µM FeCl_3_ or CuSO_4_ in modified KH buffer, using a protocol previously described for studies of Aβ [28,29]. An aliquot was taken immediately after mixing to provide the t_0_ data point, and the rest of the sample was then placed in an incubator at 37 °C inside a sealed 1.5 mL microcentrifuge tube for 7 days to provide the t_1wk_ data point. In preparation for XANES acquisition, 2.5 µL of the sample was deposited on a thin Ultralene film and allowed to dry (approximately 1 h at 37 °C). This procedure was repeated three times per spot in order to deposit enough of the sample to obtain XANES spectra in fluorescence mode with reasonable signal-to-noise (S/N) ratios. Except for the photo-reduction tests, the incoming hard X-ray beam was attenuated using 0.5 mm (when acquiring near the Cu edge) or 0.1 mm (Fe edge) of aluminum foil to minimize beam damage. XANES traces were subjected to an edge-step normalization and flattened using the standard protocol in the Athena fitting software [56].

### 2.4. Soft X-ray Spectromicroscopy

Soft X-ray spectromicroscopy was used for STXM examination of the nanoscale morphology and chemical composition of Aβ/iron structures formed in vitro. Five µL of the incubated Aβ/iron suspension (35 μM Aβ) was deposited onto 100 × 100 μm SiO_2_ membrane windows (DuneSciences; 75 nm thickness), and the excess liquid was removed with filter paper. The membranes were mounted onto STXM sample plates for X-ray spectromicroscopy. In an effort to maintain the oxidation state of iron, all sampling and mounting was performed within a nitrogen-filled glove bag, and the samples were transported to the X-ray microscope in a nitrogen-filled vessel. Prior to sample loading, the STXM end-station was purged with nitrogen. 

The STXM measurements were performed at the Swiss Light Source (Villigen, Switzerland) on the PolLux beamline using the STXM end-station. The focused X-ray spot size was approximately 20 nm. The energy-specific images were created by raster scanning the sample at the point of the focused X-ray beam and recording the intensity of the transmitted X-rays. In order to minimize photon-dose effects, thus best preserving sample chemistry during the STXM measurements, dwell (exposure) times were kept to a minimum (≤ 4 ms/point). To assess the chemical composition of Aβ/iron structures at a nanoscale spatial resolution, speciation maps were created by taking paired images: one at an energy corresponding to a feature of interest (e.g., the amide peak at the carbon *K*-edge [288.3 eV]) [57,58] and one a few eV below this feature. The off-peak image was then subtracted from the on-peak image generating an artefact-free contrast map, displaying the chemical speciation of the examined region. To provide further information regarding the chemical state of the sample material, X-ray absorption spectra were created by collecting multiple images, collectively referred to as a stack, over a desired energy range (in this case the carbon *K*-edge [280–320 eV] and the iron *L*_2,3_-edge [700–740 eV]). The transmitted X-ray absorption intensities from the stack images were converted to optical density using regions that did not contain any sample material, thereby removing background absorption features arising from the beamline. This approach to X-ray spectromicroscopy allows an X-ray absorption spectrum to be generated from every pixel of a stack image, enabling the chemical characterization of highly localized regions of interest. Carbon *K*-edge spectroscopy was performed prior to higher energy iron *L*_2,3_-edge spectroscopy to minimize X-ray induced damage to carbon structures. 

The STXM data were processed using the aXis 2000 software package (http://unicorn.mcmaster.ca/aXis2000. html). ImageJ software was used to adjust the brightness and contrast of X-ray microscopy images. (Pseudo) colored composite images were created by converting grey-scale X-ray microscopy images to false color, before recombining the images as overlays. The iron *L*_2,3_-edge X-ray absorption spectra obtained in these experiments were fitted to a series of reference iron standards (Fe(III), Fe_3_O_4_, and Fe(II), see Appendix B Figure A5) using a non-linear least squares fitting procedure to provide a quantitative estimate of the iron phases contributing to the experimental data. The reference standards were appropriately scaled using the factors determined by normalizing the X-ray absorption intensity for each reference iron phase to the integrated intensity over the iron *L*_2,3_ absorption edges, as previously described [13]. 

### 2.5. Mass Spectrometry

Mass spectrometry was performed to ascertain whether the direct binding of iron and copper ions to amyloid β and α-synuclein occurred, and if so, controlled fragmentation of the peptide backbone was used to obtain residue-specific information on the binding region(s) involved. MS analysis of the Aβ-metal and α-syn-metal complexes was performed using a 12 Tesla Bruker solariX Fourier transform ion cyclotron resonance (FTICR) instrument, using procedures which we have recently described for Aβ [59]. These samples were prepared in 20 mM aqueous ammonium acetate, following standard procedures for native MS [42,44]. The aliquots were maintained at room temperature and transferred into the gas phase by ESI after approximately 10 min. Approximately 10 µL of sample was transferred to a glass capillary that was prepared in-house. For ESI, a potential difference of 1 kV was applied using a nichrome wire inserted in the distal end of the capillary. The instrument was operated at a nominal resolving power of 400,000 at *m/z* 400, and the quadrupole mass filter of the instrument was used to select a precursor ion type for tandem MS experiments. Fragmentation was induced using either collision-induced (CID) or electron capture dissociation (ECD) depending on the precursor ion. The peak assignment tables can be found in Appendix D, and additional spectral data for the MS and X-ray results in this paper are available from the University of Warwick open access research repository (WRAP) at http://wrap.warwick.ac.uk/127048.

### 2.6. Transmission Electron Microscopy

**Figure A2:**
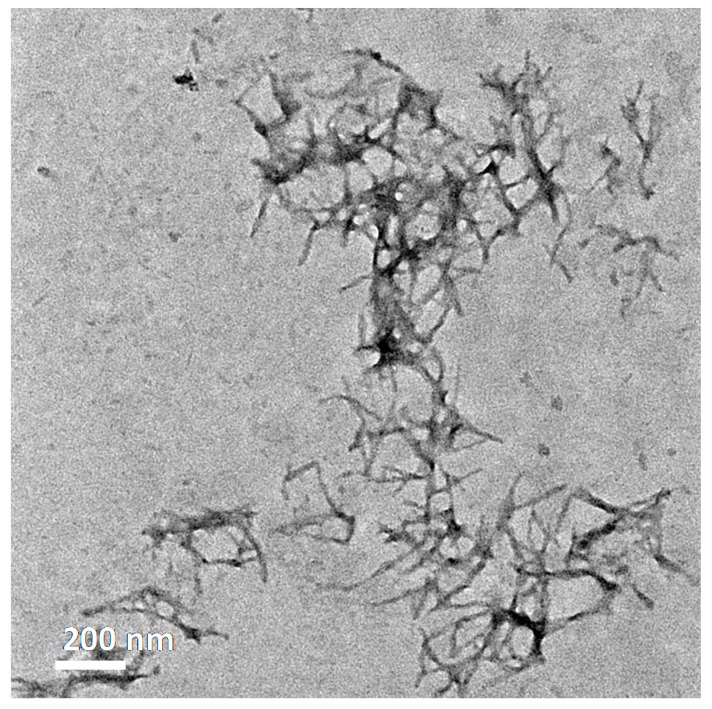
TEM image of Aβ(1–42) after incubation at 37 °C for 375 h in 100 mM aqueous ammonium acetate, at a magnification of 12000 X, using a peptide concentration of 12.5 μM and a CuSO_4_ concentration of 12.5 µM.

The supporting analysis with TEM provided high-resolution images of the peptide/metal structures analyzed by MS (Appendix A Figure A2) and X-ray spectromicroscopy (Appendix B Figure A6). TEM was performed on a parallel series of samples taken at fixed time-points from the same aliquots as those analyzed by MS. A JEOL 2011 LaB6 was used, operating at 200 kV with a GATAN ultrascan camera. Where required, uranyl acetate was used for contrast. TEM was also carried out for the samples that had already been analyzed by STXM. For this, a JEOL 1230 microscope operating at 100 kV was used, and no dyes or contrast agents were introduced. STXM was carried out prior to the TEM analysis to prevent electron beam induced changes to sample chemistry.

## 3. Results

### 3.1. Hard X-Ray Analysis

Iron and copper XANES data were acquired from a series of Aβ(1–42) and α-syn aggregates prepared in-vitro (Section 2.1 and Section 2.3), with the aliquots dried on Ultralene membranes for analysis. The iron XANES analysis was also performed on biological tissue (human brain, Section 2.2), dried onto Ultralene, using μXRF to first locate regions of interest before acquiring iron XANES scans from these sites. (In principle, copper XANES can also be acquired from brain tissue, but in practice the concentration of copper is typically an order of magnitude lower than iron, requiring an additional method optimization to acquire high-quality XANES spectra that delivers reliable insights into the copper chemistry; this is work in progress.) XANES analysis enabled the observation of a chemical reduction of the transition metals arising from two sources: As a result of photo-reduction where the beam exposure was not controlled, and also arising from the incubation of the metals with aggregating peptides. 

In preparation for the XANES measurements, we determined the extent to which it was necessary to control photo-reduction from X-ray beam exposure was determined. Appendix C Figure A7 shows, for in-vitro prepared α-synuclein with Cu(II), how scan reproducibility and features are increasingly preserved as the thickness of aluminum foil used upstream to attenuate the incident beam is increased. The preservation of spectra within the experimental error was observed with 0.5 mm aluminum attenuation at room temperature and ambient pressure, and sample homogeneity was also confirmed. This level of attenuation was demonstrated, by the use of repeated scanning, to mitigate photo-reduction for all four experiment conditions, and 0.5 mm Al attenuation was used in all subsequent measurements of copper XANES. The equivalent level of attenuation required at the iron edge was 0.1 mm Al. 

#### 3.1.1. XANES of Iron in Human Brain Tissue

In order to perform XANES of iron from the sites of interest in human brain tissue, μXRF analysis was first used to make multi-metal maps over the regions of human brain tissue. XANES analysis was then performed to investigate the chemical state of the metals. In the example shown in Figure 1, substantia nigra tissue (donated post-mortem from a confirmed case of Parkinson’s disease) retained some neuromelanin-pigmented cell bodies (and in some cases neuromelanin released into the extra-cellular matrix), showing elevation of iron, copper, and zinc levels relative to the surrounding tissue. This example is provided to showcase μXRF analysis of metal distribution in tissues, aided by the endogenous contrast from the pigmented neuromelanin which provides information about the tissue structure. These regions of concentrated metals map to the distribution of pigmentation and are consistent with the high affinity of neuromelanin for transition metals which may impact neuronal vulnerability [37,38,39].

The beam exposure at each pixel during XRF mapping was short (<1 s), but there is scope when performing subsequent XANES at the sites of interest (e.g., at the inset region outlined in Figure 1a) for photo-reduction of the metals to occur. The repeat scans using 0.1 mm Al attenuation were performed at the site of interest shown in Figure 1a, demonstrating that XANES at the iron K-edge was unchanged within experimental error (<2%). The fitting of the repeat-scanned region in the NM-rich neuron (blue and orange traces in Figure 1e) with FeCl_3_ (shown as a solid black line) supports the interpretation that NM-bound iron is predominantly in ferric form in these pigmented regions. This finding is supported by prior reports [34].

This example of how μXRF can be used to locate transition metal ion distributions in tissues is facilitated by a choice of a sample with natural contrast arising from the NM pigment. For μXRF analysis in this energy range, supporting techniques are required to correlate information about other organic constituents, including protein aggregates, with the metal ion distributions, for example, optical microscopy with the introduction of stains where necessary. The sections used for μXRF cannot be stained in advance because for μXRF, staining would introduce an unacceptable level of contamination.

#### 3.1.2. XANES of Iron and Copper in In-Vitro Systems

**Figure 2:**
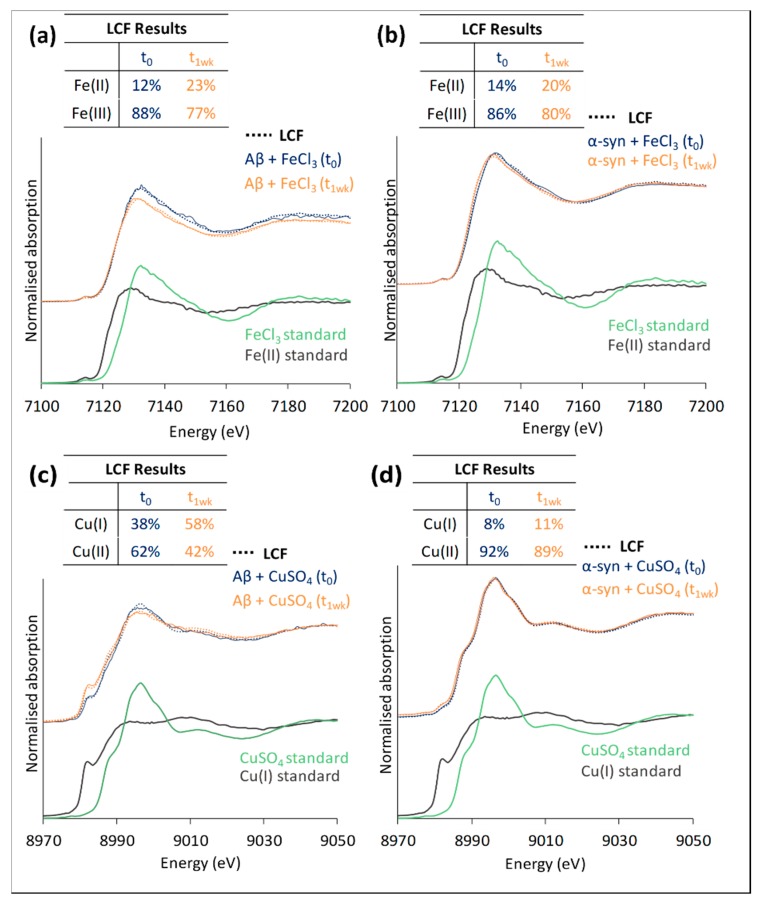
XANES traces showing the effect of 1 week’s incubation on metal oxidation states. (**a**) Aβ and (**b**) α-syn on the oxidation state of Fe, and (**c**) Aβ and (**d**) α-syn on the oxidation state of Cu. Ferric and ferrous standards (Fe(III)Cl_3_ and [Fe(II) + 1,10-phenanthroline], respectively) were used for linear combination fitting (LCF) of the spectra in (**a**) and (**b**), and cupric and cuprous standards (Cu(II)SO_4_) and [Cu(I)-glutathione] were used for (**c**) and (**d**); the results from the LCF are tabulated in each panel; below the experimentally-acquired XANES spectra are the reference standards used to fit them for each time point (**a**)–(**d**). In (**a**) and (**b**), inclusion of a small contribution from iron(III) ammonium citrate in the LCF resulted in improved quality of fit. χ^2^ values are as follows for t_0_ and t_1wk_ fits, respectively: (**a**) 0.06, 0.03; (**b**) 0.04, 0.02; (**c**) 0.07, 0.06; (**d**) 0.002, 0.04. Traces for metal-peptide incubations are vertically offset from the reference standards for clarity.

XANES from aliquots of the iron or copper co-incubated with amyloid β or α-synuclein was performed with the attenuated beam protecting against photo-reduction. XANES analysis of these in-vitro formed aggregates revealed a marked chemical reduction of iron and copper arising from the co-incubation with Aβ, and to a lesser extent with α-syn, as shown in Figure 2.

A comparison of the four in-vitro systems (Fe or Cu plus Aβ or α-syn) indicated that the increased tendency for chemical reduction under incubation conditions observed for Aβ was mirrored by the extent to which photo-reduction arose with the un-attenuated beam. There is evidence that these same processes arise in mammalian brain tissue [13,14], and in this context, it is important to confirm the underlying mechanism(s) responsible for the observed chemical reduction of these metal species.

### 3.2. Nanoscale Chemical Speciation of Aβ/Iron Aggregate Structures Formed In-Vitro

Having surveyed larger sample areas (~mm^2^) with XRF in Section 3.1, using XANES to gain an overview of dominant inorganic phases at a cellular resolution, soft X-ray spectromicroscopy techniques complemented by transmission electron microscopy were used to probe intact protein/metal structures at length scales spanning tens of nanometers to microns. 

#### 3.2.1. Amyloid β(1–42) and Iron(III) In-Vitro

**Figure 3:**
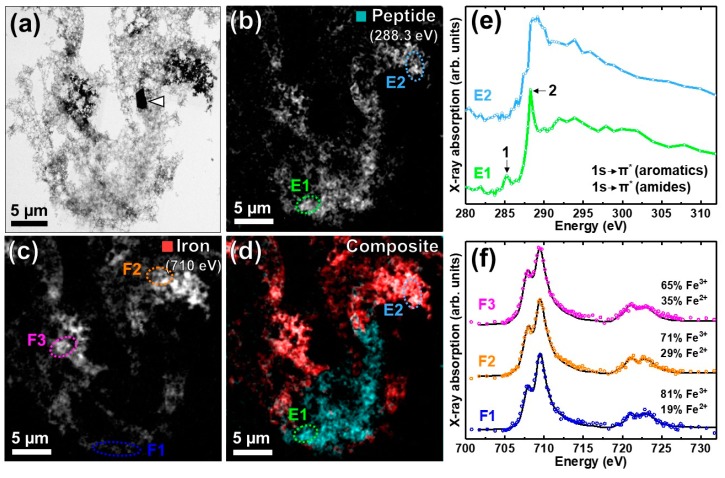
TEM and STXM analysis of an Aβ/iron aggregate formed in-vitro. (**a**) TEM image. Arrowhead shows a crystalline artefact originating from the buffer medium. (**b**) Carbon *K*-edge peptide speciation map. (**c**) Iron *L*_3_-edge speciation map. (**d**) Composite image displaying peptide (cyan) and iron (red) content of the aggregate. **(e)** Carbon *K*-edge X-ray absorption spectra from the aggregate regions highlighted in (**b**) and (**d**). (**f**) Iron *L*_2,3_-edge X-ray absorption spectra (colored circles) from the aggregate regions highlighted in (**c**). The solid lines for the spectra correspond to the best fit curve created by superposition of suitably scaled iron reference X-ray absorption spectra.

The morphology, spatial distribution of chemical elements, and the composition of aggregate structures formed through the co-incubation of Aβ(1–42) and iron(III) in vitro were determined using STXM and TEM (Figure 3). TEM (Figure 3a) revealed aggregate morphology that is largely fibrillar, approximately 25 µm in diameter and of varying electron density, containing multiple electron opaque regions (see Appendix B Figure A6 for high magnification TEM images). To establish the chemical composition of the aggregate at a nanoscale spatial resolution, STXM speciation maps were collected at the carbon *K*-edge (to visualize peptide (Aβ) content) and the iron *L*_3_-edge. Carbon *K*-edge mapping (Figure 3b) showed that peptide distribution closely follows the aggregate morphology under TEM (Figure 3a), demonstrating the aggregate to be composed of Aβ. Iron *L*_3_-edge mapping (Figure 3c) showed multiple regions of the iron accumulation within the aggregate, suggesting the co-deposition of Aβ and iron. By comparing the TEM images with the STXM iron speciation map, it is apparent that the most electron dense regions in Figure 3a correspond to regions of high iron loading within the aggregate (Figure 3c). It is interesting that in mouse models of amyloid over-production (e.g., APP/PS1), there is evidence of a direct correspondence between amyloid deposition and iron deposition [14], while the distribution of iron in human amyloid deposits is far more heterogeneous [13,16]. Our findings presented here are consistent with the prior work on human amyloid plaque material.

#### 3.2.2. STXM Spectromicroscopy of Aβ(1–42) and Iron(III) In Vitro

To examine the organic composition of the Aβ/iron aggregate in more detail, STXM measurements were performed over the entire carbon *K*-edge (280–320 eV). The resulting X-ray absorption spectra obtained from the examined areas (highlighted in Figure 3b,d) are displayed in Figure 3e. Spectrum E1 was obtained from a region of the aggregate containing little to no iron, and comprised two sharp peak features at 285.2 eV and 288.3 eV, corresponding to the 1s-to-π* transitions of peptide aromatic and amide groups respectively [58]. Conversely, spectrum E2 was acquired from a region of the aggregate containing a high level of iron. By comparing spectrum E2 to E1, a suppression of the 285.2 eV peak can be seen, along with a broadening of the sharp 288.3 eV feature. These results suggest that the alterations to Aβ organic composition may occur upon iron loading.

Further STXM measurements were performed over the iron *L*_2,3_-edge to determine the chemical composition of the iron within the aggregate. The relative proportion of iron phases contributing to each iron *L*_2,3_-edge absorption spectrum was calculated using a non-linear least-squares fitting procedure, as previously described [13]. The reference iron spectra are displayed in Appendix B Figure A5 Iron *L*_2,3_-edge X-ray absorption spectra from three regions of the aggregate (Figure 3c) are shown in Figure 3f. The fitting of the spectrum obtained from region F1 showed this spectrum to be primarily (81%) composed of Fe(III) with a minor (19%) contribution from Fe(II). The ferric content of this region is evidenced by the presence of a low energy peak at 708 eV followed by a dominant 709.5 eV peak at the *L*_3_-absorption edge both arising from Fe(III) cations (Appendix B Figure A5) [60]. The fitting of the X-ray absorption spectra from regions F2 and F3 also showed Fe(III) to be the primary component, but with evidence of increased Fe(II) content (29% and 35% respectively) compared to region F1. This was made apparent by an enhancement in the intensity of the *L*_3_-edge peak feature at 708 eV, the principal Fe(II) absorption peak (Appendix B Figure A5) [60], with respect to the Fe(III) feature at 709.5 eV. 

Taken together, these X-ray spectromicroscopy results demonstrate the sensitivity of STXM to spatial and chemical composition, allowing the detection of localized changes.

### 3.3. Mass Spectrometry Allowed Determination of the Specific Binding Regions in [α-syn + Cu], [α-syn + Fe], [Aβ + Cu], and [Aβ + Fe] Complexes

Having used X-ray methods to image chemical properties on the micro- and nanoscale, the series of peptide/metal mixtures examined by XANES was analyzed using mass spectrometry (MS) to determine the regions on the peptides that bind the transition metals Cu and Fe. 

**Figure 4:**
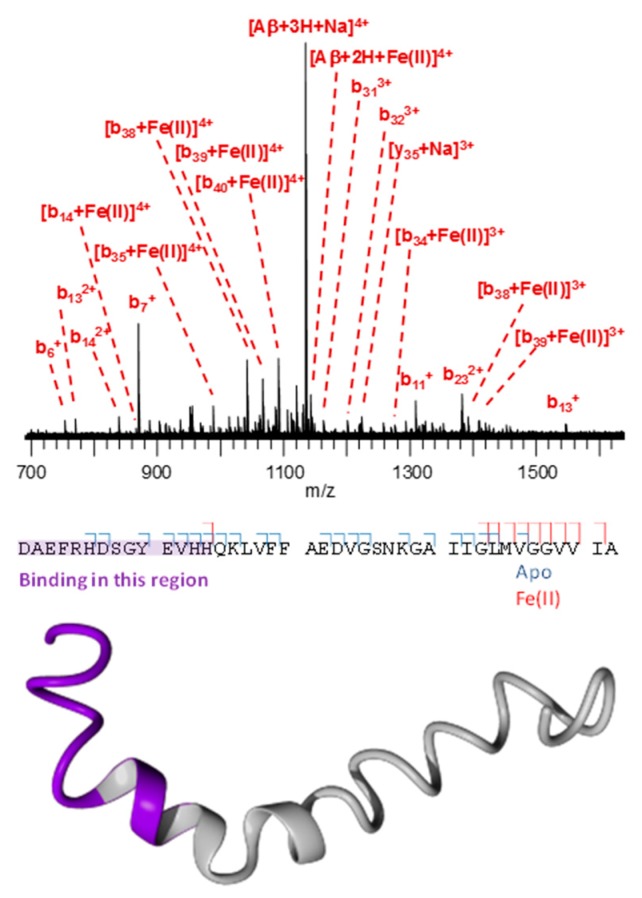
Collision-induced (CID) fragmentation of [Aβ + 2H + Fe(II)]^4+^. The fragments are summarized below the mass spectrum, and the possible binding region is indicated. A 3D structural representation of Aβ(1–42) is shown at the bottom, with the possible binding region colored purple.

A 1:1 complex of Aβ and Fe(II) was generated in an aqueous solution containing 12.5 µM peptide, 200 µM FeCl_2_, and 20 mM ammonium acetate, with 400 µM hydroxylamine hydrochloride added to protect Fe(II) from the oxidation by atmospheric air. The resulting 1:1 complex was detected by MS, as a peak corresponding to a mass increase of 53.939 Da (4565.209 Da compared to 4511.270 Da), matching the replacement of two protons with Fe, was observed at approximately 10% of the intensity of the peak corresponding to the apo-peptide. The [Aβ + 2H + Fe]^4+^ charge state was selected (*m/z* 1142.305) and interrogated with CID using a potential offset of 30 V. Due to the limited resolution of the quadrupole mass filter, some co-isolation of [Aβ + 3H + Na]^4+^ at *m/z* 1134.320 occurred. As this adduct seems somewhat more stable than the Fe adduct toward collisional activation, the signal due to the Na adduct is abundant in the fragmentation spectrum (Figure 4). However, due to the high mass accuracy and resolving power of FTICR-MS, this does not interfere with the detection and assignment of Fe-containing fragments.

Following a similar methodology as for Aβ, a 1:1 complex of Fe(II):α-syn was detected by ESI-MS in a solution containing 7 µM protein, 140 µM FeCl_2_, and 280 µM hydroxylamine hydrochloride. This resulted in the observation of the adduct with a signal intensity of approximately 35% of that associated with the apo-protein. The [α -syn + 11H + Fe]^13+^ charge state of this complex was then selected (*m/z* 1116.787) and interrogated with the collision-induced dissociation using a potential offset of 16 V.

**Figure 5:**
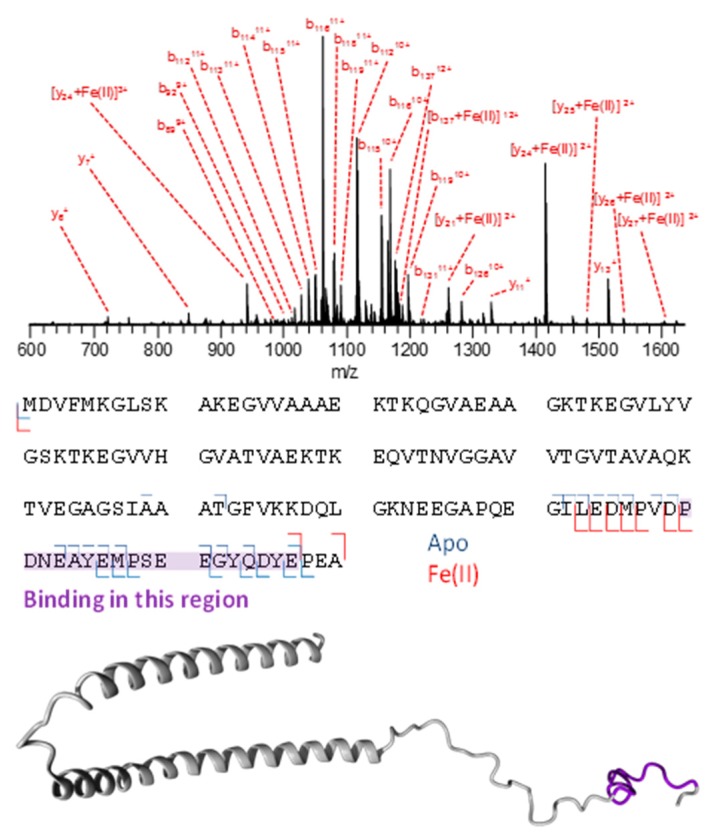
CID fragmentation of [α-syn + 11H + Fe(II)]^13+^. The fragments are summarized below the mass spectrum, and the possible binding region is indicated. A 3D structural representation of α-synuclein is shown at the bottom, with the possible binding region colored purple.

The presence of an Fe(II)-containing N-terminal *b_137_* fragment in the CID spectrum of [α-syn + 11H + Fe(II)]^13+^ provides evidence that the binding site is located within the first 137 amino acid residues (Figure 5). Meanwhile, the smallest C-terminal fragment carrying the metal is *y_21_*, indicating a binding site within the last 21 residues. Combined, these results indicate that the binding region is located in the P(120)DNEAYEMPSEEGYQDYE(137) stretch of the protein. 

**Figure A3:**
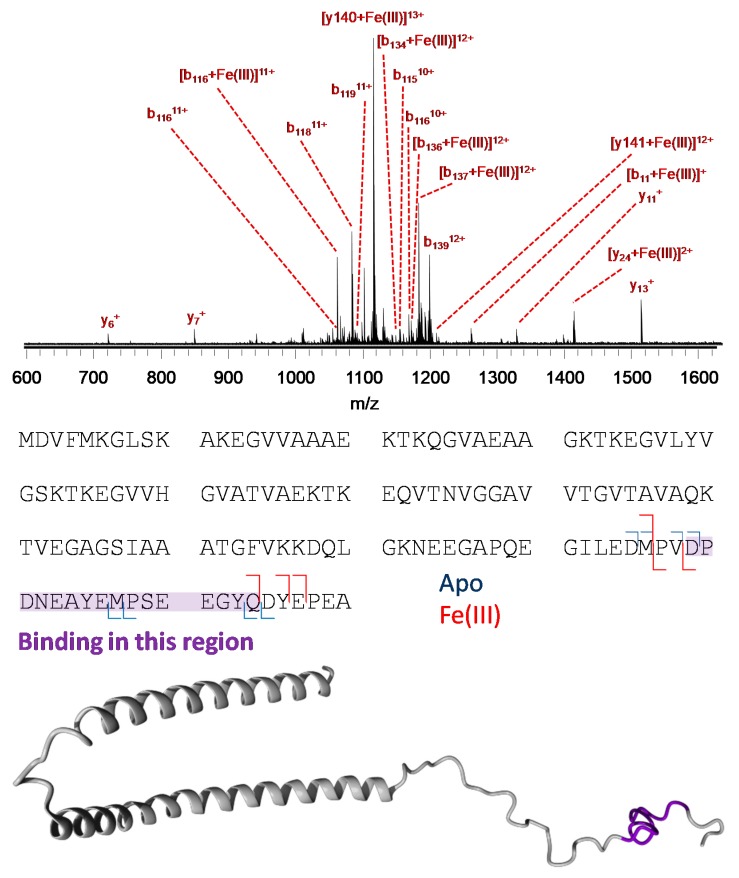
Tandem MS analysis of the [α-syn + Fe(III)] complex, showing results from CID fragmentation of [α-syn + 13H + Fe(III)-NTA^3-^]^13+^. The fragments are summarized below the mass spectrum, and the possible binding region is indicated. A 3D structural representation of α-syn is shown at the bottom, with the possible binding region colored purple. Fe(III) was added as a 1:1 complex with NTA^3-^ to stabilize it towards hydrolysis, precipitation, and aggregation at pH 7. The ligand is lost before backbone fragmentation occurs, and hence metal-bound fragments are detected carrying the free Fe(III) cation.

Similarly, the CID spectrum of [α-syn + 13H + Fe(III)-NTA3-]^13+^ (Appendix A Figure A3) shows that the binding region for the 3+ oxidation state of iron was very similar, i.e., D(119)PDNEAYEMPSEEGYQ(134). Based on these results, it is likely that coordination in both cases involves the side-chains of the D, E and/or Y residues present in this region, as suggested by other methods [18,61]. 

**Figure A4:**
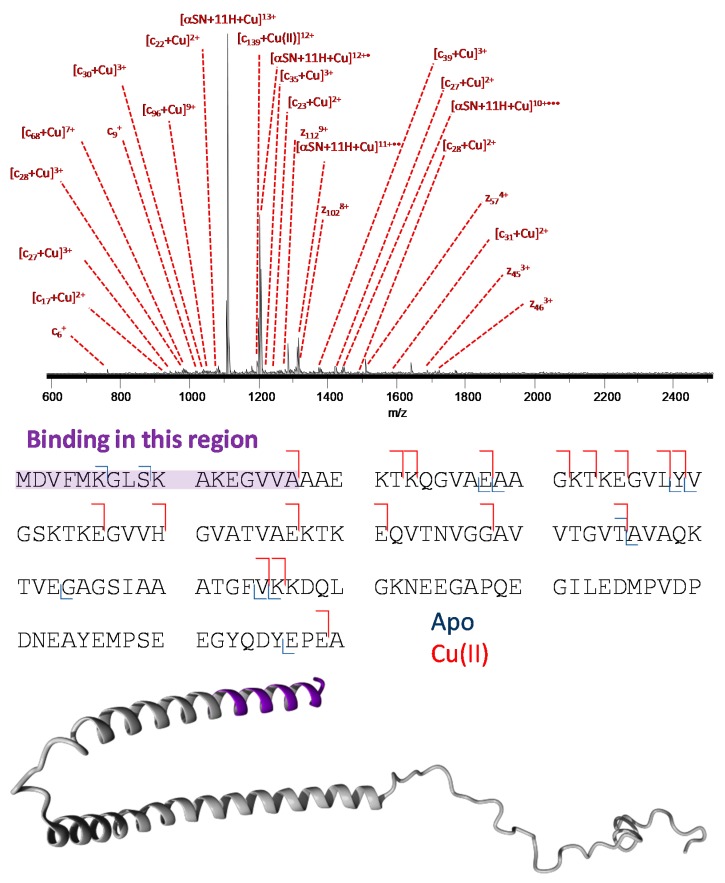
Tandem MS analysis of the [α-syn + Cu(II)] complex, showing the results from electron capture dissociation (ECD) fragmentation of [α-syn + 11H + Cu(II)]^13+^. The fragments are summarized below the mass spectrum, and the possible binding region is indicated. A 3D structural representation of α-syn is shown at the bottom, with the possible binding region colored purple.

**Figure A5:**
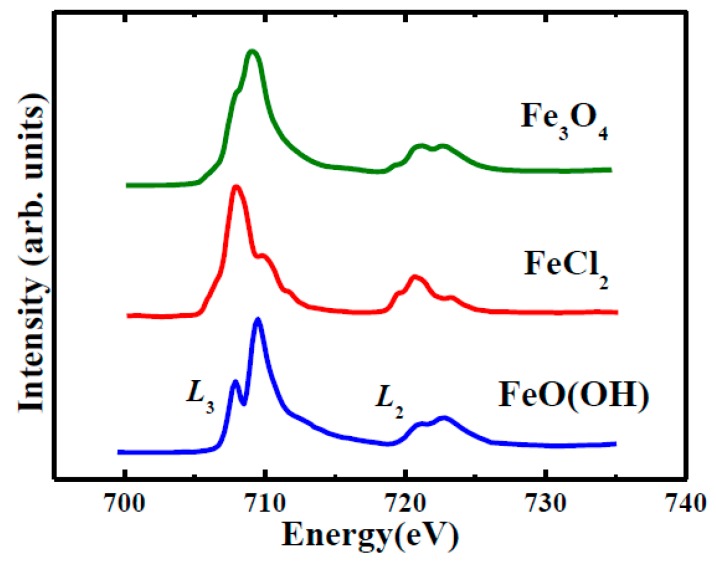
Reference iron L_2,3_-edge X-ray absorption spectra for ferric (FeO(OH); blue), ferrous (FeCl_2_; red) and magnetite (Fe_3_O_4_; green) iron phases.

**Figure A6:**
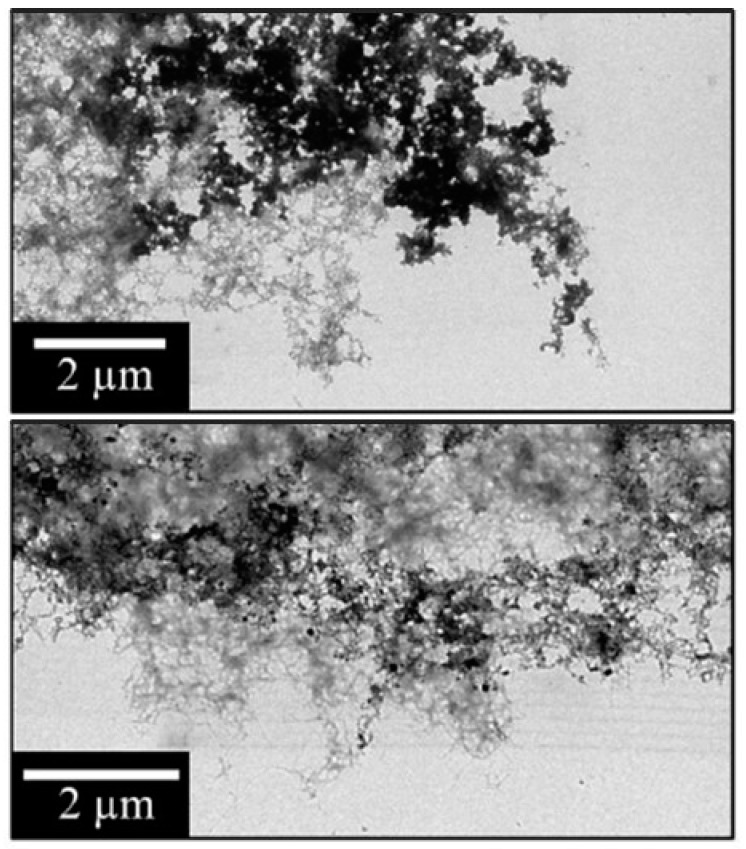
Additional high magnification TEM images from the aggregate shown in Figure 3.

**Figure A7:**
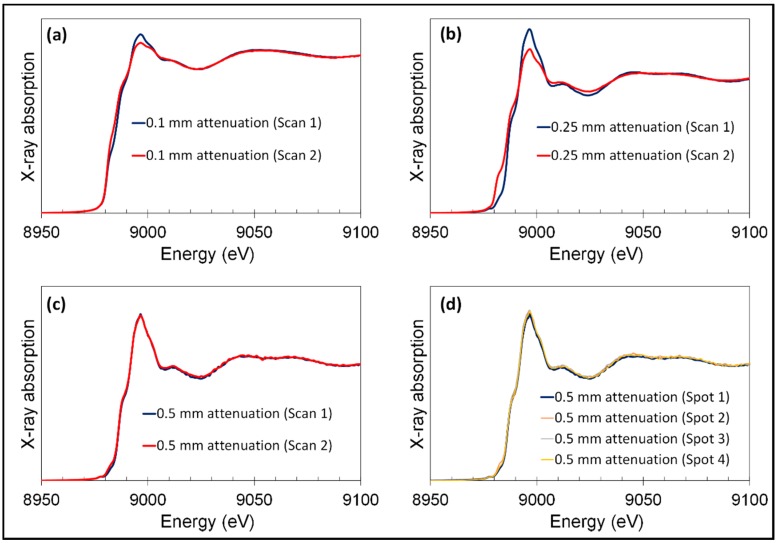
Optimizing measurement conditions for XANES collected from α-syn + Cu(II)SO_4_ at t_0_. Repeat scans using (**a**) 0.1 mm Al attenuation, (**b**) 0.25 mm Al attenuation (**c**) 0.5 mm Al attenuation. Note that for the highest photon flux condition (**a**), the successive scans are similar as a consequence of significant photo-reduction affecting both scans; (**d**) XANES from multiple spots on the same sample demonstrate homogeneity, with the results of the linear combination fitting matching to within 3% between the four sites (spot 1—spot 4) sampled at ~100 μm apart).

The presence of an N-terminal *c_17_* fragment bound to copper in the ECD spectrum of [α-syn + 11H + Cu(II)]^13+^ (Appendix A Figure A4) shows that the highest-affinity binding region for this metal is located in the 17 residues closest to the N-terminus, i.e., M(1)DVFMKGLSKAKEGVVA(17). 

A *b_14_* fragment carrying Fe(II) is observed in the CID spectrum of [Aβ + 2H + Fe(II)]^4+^ (Figure 4) indicating that binding occurs in the D(1)AEFRHDSGYEVHH(14) stretch of the peptide. This interaction is most likely mediated through the histidine residues in this region. In contrast to α-syn, which was found to bind iron and copper in two distinct sequence regions, both Fe(III) and Cu(II) bind to Aβ in a very similar region to Fe(II) as the authors have shown recently [59], which is consistent with prior evidence in the literature [18,61,62,63,64]. 

## 4. Discussion

The complexes of Aβ(1–42) or α-syn with copper or iron were studied using XANES, STXM, and MS. XANES analysis of the four complexes prepared in vitro (Fe(III) or Cu(II) co-incubated with Aβ(1–42) or α-syn), showed that X-ray-beam-induced photo-reduction was successfully mitigated with appropriate beam attenuation prior to the final series of measurements on previously unanalyzed regions of each sample. It was necessary to test for optimal conditions prior to each measurement, as excessive beam attenuation compromised S/N ratios. As noted in the results, XANES experiments conducted under these controlled conditions revealed that the chemical reduction of ferric iron and cupric copper arose when each metal was incubated in the presence of Aβ(1–42) for one week, and to a lesser extent for ferric iron incubated with α-syn. Under these conditions, Aβ(1–42) appeared to have a greater reductive capacity than α-syn. The ease with which the photo-reduction of the metals could be achieved paralleled the extent to which the chemical reduction of the metals arose during incubation with the peptides. This raises the possibility that the mechanism(s) responsible for the chemical reduction of Fe or Cu co-incubated with Aβ(1–42), and to an extent with α-syn, are amplified with X-ray beam exposure.

STXM examination of aggregates formed via the co-incubation of Aβ(1–42) and Fe(III) in vitro, performed at the carbon *K* and iron *L*_3_-absorption edges, explored multiple regions of iron co-precipitation in a peptide-dense aggregate structure of approximately 25 µm in diameter. The heterogeneity of iron loading was evident, and in the regions of iron loading, peptide and iron distribution were closely correlated, indicating that iron was incorporated into the aggregating Aβ(1–42) structure. TEM imaging performed subsequent to STXM analysis appeared to confirm this correlation of Aβ(1–42) and iron distribution, with iron-loaded regions presenting as electron-dense fibrillar structures within the Aβ aggregate.

By performing STXM measurements across the entire carbon *K*-edge, the organic composition of Aβ was shown to be altered dependent on iron co-localization. In aggregate regions devoid of iron, Aβ provided carbon *K*-edge absorption spectra consistent with the theoretical spectrum for Aβ(1–42) (see Figure 2 in [28]) and albumin peptide references [14]. However, in aggregate regions containing high levels of iron, an altered spectrum was recorded, displaying a suppressed aromatic absorption peak, and a broadened amide absorption peak.

An examination of the aggregate iron across the iron *L*_2,3,_-absorpotion edge showed a spatially-dependent variation in the iron oxidation state. Iron *L*_2,3_-edge X-ray absorption spectra from all three regions were found to primarily comprise Fe^3+^ with more minor contributions from Fe(II) cations. The Fe(II) content was seen to vary from 19% to 35% across the different regions, again demonstrating a heterogeneity in sample chemistry within an individual aggregate structure. 

These findings demonstrate the power of STXM for the examination of heterogeneous sample materials on a spatial scale relevant to pathological lesions commonly found in neurodegenerative disorders (typically <20 µm). Whilst only the carbon and iron content of Aβ/iron structures were examined in this instance, the large operational energy range typically offered by STXM microscopes allows for the examination of absorption edges throughout the water window (e.g., C, N, O and P), and multiple metals (such as Fe, Cu and Ca) implicated in the development of neurodegenerative disorders [65]. This chemical sensitivity of STXM combined with nanoscale spatial resolution, enables the distribution and chemical speciation of protein/metal structures to be realized to an extremely high level of detail. Importantly, these qualities allow the identification of localized (sub-micron) changes to both organic and inorganic sample chemistry that would not be revealed by an analysis on the microscale. 

Mass spectrometry of the in-vitro series of Aβ(1–42) or α-syn, incubated with copper or iron, confirmed that both iron and copper were coordinated by the histidine-rich N-terminal domain of Aβ, but bound to different regions in α-syn. In α-syn, iron was coordinated by the C-terminal domain, which is rich in aspartic and glutamic acid residues. Copper(II) preferred the N-terminal domain, and may be coordinated by the thioether sulfur of the two methionine residues in this region [18,66]; an alternative lower-affinity site for Cu(II) is near the C-terminus. As highlighted in Figure 2, a small but consistent improvement in the χ^2^ value obtained with the linear combination fitting of the XANES spectra was observed if, in addition to iron(III) chloride, a minor contribution of iron(III) ammonium citrate (approximately 40% of that of FeCl_3_) was included as a standard. This can be rationalized in light of our MS results, as Fe(III) in the citrate complex is coordinated by oxygen ligands, similar to the coordination by nitrogen (histidine; in Aβ) or oxygen (aspartic/glutamic acid; in α-syn) ligands in the peptide/protein complexes. These results showcase the power of native top-down MS [53] to investigate peptide-metal complexes on a molecular scale, as the authors have recently explored in-depth [59]. Importantly, in these experiments, extremely small sample amounts (picomoles) were sufficient to obtain high-quality (tandem) MS data.

The precise relationship between the metal coordination and reductive capacity of these species is still under investigation. As Fe(II) and Cu(I) can easily re-oxidize in aqueous solution, it can be envisaged that the redox chemistry of these complexes can drive oxidative stress and neuron damage in the proteinopathies. Improving the understanding of the bioinorganic chemistry of these molecules may create opportunities to improve the treatment strategies for this class of neurodegenerative disorders.

In conclusion, elucidating the chemistry of these metal-biomolecule complexes is critical for understanding the etiology of neurodegenerative diseases, and this improved understanding may, in the longer term, open up new avenues for treatment.

## Figures and Tables

**Table 1:** Summary of primary techniques.

Technique	Abbreviation	Description
Mass Spectrometry	MS	Electrospray ionization (ESI) tandem MS is used to determine regions on peptides where metals bind, by analyzing the molecular masses of fragments where the amide backbone of the peptide has been cleaved while preserving protein-metal interactions. The fragmentation pattern indicates the binding region(s), as the fragments containing the residue(s) that interact(s) with the metal show a characteristic mass increase.
Scanning Transmission X-ray Microscopy	STXM	Synchrotron soft X-ray microscopy is used in transmission mode to obtain images at tens of nanometer spatial resolution, acquired sequentially in stacks as a function of energy. These data contain spectral information about the chemistry of each region of interest selected within the image.
X-ray Absorption Near-Edge Spectroscopy	XANES	Synchrotron hard X-ray microscopy is used in fluorescence mode to obtain energy scans from elements of interest, where the structure of the spectrum is sensitive to the local chemical environment of the scattering element.
Transmission Electron Microscopy	TEM	Electron beam imaging is used to investigate the forms of peptide aggregate present in the samples analyzed by MS, STXM, and XANES.

## Appendix D. Mass Spectrometry Peak Assignment Tables

**Table A1:** Collision-induced dissociation of [α-syn + Fe(III)-NTA].

Ion	Exact *m/z*	Observed *m/z*	Error (ppm)
[y6]^+^	723.2832	723.2835	0.3
[y7]^+^	851.3418	851.342	0.3
[b116]^11^+	1062.6557	1062.6562	0.4
[b116+Fe(III)]^11+^	1067.4659	1067.4663	0.4
[b118]^11+^	1080.4849	1080.4854	0.4
[b119]^11+^	1090.9419	1090.9433	1.2
[y140+Fe(III)]^13+^	1116.7096	1116.71	0.3
[b134+Fe(III)]^12+^	1149.4952	1149.4948	−0.4
[b115]^10+^	1155.7165	1155.7171	0.5
[b116]^10+^	1168.8206	1168.8209	0.3
[b136+Fe(III)]^12+^	1172.6694	1172.6705	0.9
[b137+Fe(III)]^12+^	1183.4229	1183.4232	0.2
[y22+Fe(III)]^2+^	1315.9455	1315.9458	0.2
[y24+Fe(III)]^2+^	1414.0061	1414.0067	0.4
[y13]^+^	1513.5966	1513.5967	0.1
[y14]^+^	1644.6371	1644.6362	−0.5
Average			0.32
Standard Dev			0.41

**Table A2:** Collision-induced dissociation of [α-syn + Fe(II)].

Ion	Exact *m/z*	Observed *m/z*	Error (ppm)
[y3]^+^	316.1504	316.1504	0.3
[y4]^+^	445.193	445.1929	−0.2
[y6]^+^	723.2832	723.2834	0.3
[y7]^+^	851.3418	851.3426	1.0
[y24+Fe(II)]^3+^	943.3424	943.343	0.6
[b89]^9+^	981.2003	981.2009	0.6
[b118]^12+^	990.5285	990.5304	2.0
[b92]^9+^	1008.2138	1008.215	1.2
[b112]^11+^	1018.2745	1018.2755	1.0
[b113]^11+^	1028.5548	1028.5555	0.7
[b114]^11+^	1040.286	1040.2868	0.8
[b115]^11+^	1050.743	1050.7432	0.2
[b116]^11+^	1062.6557	1062.6561	0.3
[y9]^+^	1071.4266	1071.4268	0.1
[b127]12^+^	1079.2265	1079.2263	−0.2
[b118]11^+^	1080.4849	1080.4851	0.1
[b119]11^+^	1090.9419	1090.9421	0.1
[y28+Fe(II)]^3+^	1106.0738	1106.0726	−1.0
[b111]^10+^	1108.6928	1108.6937	0.8
[y140]^13+^	1112.6395	1112.6405	0.9
[b140+Fe(II)]^13+^	1115.4017	1115.4025	0.7
[y140+Fe(II)]^13+^	1116.7872	1116.7873	0.1
[b112]^10+^	1120.0012	1120.0012	0.0
[b113]^10+^	1131.3096	1131.3099	0.3
[b123]^11+^	1132.3206	1132.3218	1.1
[b124]^11+^	1138.7785	1138.7784	−0.1
[b114]^10+^	1144.2139	1144.2139	0.0
[b125]^11+^	1153.6025	1153.6022	−0.2
[b115]^10+^	1155.7165	1155.7166	0.0
[b126]^11+^	1165.3336	1165.3337	0.1
[b116]^10+^	1168.8206	1168.8206	0.0
[b137]^12+^	1179.0136	1179.0136	0.0
[b137+Fe(II)]^12+^	1183.5069	1183.5072	0.2
[b118]^10+^	1188.4327	1188.4326	−0.1
[b119]^10+^	1199.9354	1199.9355	0.0
[b131]^11+^	1217.4436	1217.4437	0.0
[b132]^11+^	1222.6274	1222.6259	−1.2
[b134]^11+^	1249.093	1249.0937	0.6
[b124]^10+^	1252.5556	1252.5544	−1.0
[y21+Fe(II)]^2+^	1258.9359	1258.9364	0.4
[b126]^10+^	1281.7662	1281.7663	0.0
[b127]^10+^	1294.8703	1294.8713	0.8
[b116]^9+^	1298.5776	1298.578	0.3
[y22+Fe(II)]^2+^	1316.4494	1316.4494	−0.1
[y24+Fe(II)]^2+^	1414.51	1414.5111	0.8
[y25+Fe(II)]^2+^	1480.0303	1480.0317	1.0
[y13]+	1513.5966	1513.5984	1.2
[y26+Fe(II)]^2+^	1537.5437	1537.5462	1.6
[y27+Fe(II)]^2+^	1602.065	1602.0663	0.8
[y14]^+^	1644.6371	1644.6396	1.5
[y15]^+^	1773.6796	1773.679	−0.3
Average			0.36
Standard Dev			0.63

**Table A3:** Collision-induced dissociation of [Aβ + Fe(II)].

Ion	Exact *m/z*	Observed *m/z*	Error (ppm)
[b6]^+^	756.3424	756.3424	0.0
[b13]^2+^	772.3317	772.3321	0.5
[b14]^2+^	840.8612	840.8609	−0.3
[b14+Fe(II)]^2+^	867.8208	867.8203	−0.6
[b7]^+^	871.3694	871.3693	−0.1
[b15]^2+^	904.8904	904.8903	−0.1
[b33]^4+^	914.4486	914.4484	−0.3
[b23]^3+^	920.0930	920.0917	−1.5
[b33+Fe(II)]^4+^	927.9284	927.9271	−1.4
[b24]^3+^	953.1158	953.1152	−0.7
[b34+Fe(II)]^4+^	956.1994	956.1987	−0.8
[b16]^2+^	968.9379	968.9357	−2.3
[b25]^3+^	972.1230	972.1219	−1.1
[b35+Fe(II)]^4+^	988.9595	988.9590	−0.5
[b36+Fe(II)]^4+^	1013.7267	1013.7269	0.3
[b37+Fe(II)]^4+^	1027.9820	1027.9809	−1.1
[b38+Fe(II)]^4+^	1042.2374	1042.2368	−0.6
[b39+Fe(II)]^4+^	1067.0045	1067.0040	−0.5
[b18]^2+^	1075.0142	1075.0129	−1.1
[b28]^3+^	1081.8463	1081.8454	−0.8
[b40+Fe(II)]^4+^	1091.7716	1091.7710	−0.5
[b41+Fe(II)]^4+^	1120.0426	1120.0429	0.3
[b30]^3+^	1124.5325	1124.5312	−1.2
[Aβ+3H+Na]^4+^	1134.3202	1134.3200	−0.2
[b42+Fe(II)]^4+^	1137.8019	1137.8008	−1.0
[Aβ+2H+Fe(II)]^4+^	1142.3045	1142.3048	0.2
[b19]^2+^	1148.5484	1148.5461	−2.0
[b31]^3+^	1162.2272	1162.2275	0.3
[b10]^+^	1178.4862	1178.4860	−0.2
[b32]3^+^	1199.9219	1199.9220	0.1
[y35+Na]^3+^	1221.9705	1221.9705	0.0
[b33+Fe(II)]^3+^	1236.9021	1236.9009	−1.0
[b34]^3+^	1256.6237	1256.6254	1.3
[b34+Fe(II)]^3+^	1274.5968	1274.5957	−0.9
[b11]^+^	1307.5288	1307.5297	0.7
[b35+Fe(II)]^3+^	1318.2770	1318.2779	0.7
[b22]^2+^	1322.1224	1322.1225	0.0
[b36]^3+^	1333.3267	1333.3287	1.5
[b23]^2+^	1379.6359	1379.6386	2.0
[b38+Fe(II)]^3+^	1389.3141	1389.3165	1.7
[b12]^+^	1406.5972	1406.5977	0.3
[b39+Fe(II)]^3+^	1422.3369	1422.3377	0.6
[b24]^2+^	1429.1701	1429.1705	0.2
[b40+Fe(II)]^3+^	1455.3597	1455.3625	1.9
[b13]^+^	1543.6561	1543.6578	1.1
Average			−0.16
Standard Dev			0.97

**Table A4:** Electron-capture dissociation of [α-syn + Cu(II)].

Ion	Exact *m/z*	Observed *m/z*	Error (ppm)
[c6]^+^	769.3736	769.3736	0.0
[c9]^+^	1026.5111	1026.5095	1.6
[c17+Cu(II)]^2+^	935.4487	935.4486	0.1
[c22+Cu(I)]^2+^	1186.0824	1186.0817	0.5
[c28+Cu(I)]^2+^	1492.2439	1492.2419	1.4
[c31+Cu(I)]^2+^	1591.7918	1591.7892	1.6
[c23+Cu(II)]^3+^	833.4197	833.4196	0.2
[c28+Cu(II)]^3+^	994.8291	994.8276	1.5
[c31+Cu(II)]^3+^	1061.1943	1061.1922	2.0
[c33+Cu(I)]^3+^	1137.9112	1137.9096	1.4
[c35+Cu(I)]^3+^	1223.6237	1223.6213	1.9
[c38+Cu(I)]^3+^	1313.3483	1313.3462	1.6
[c39+Cu(I)]^3+^	1367.7028	1367.7026	0.1
[c46+Cu(I)]^3+^	1610.8368	1610.8379	−0.7
[c38+Cu(I)]^4+^	985.2631	985.2613	1.8
[c39+Cu(I)]^4+^	1026.0289	1026.0269	1.9
[c50+Cu(II)]^5+^	1045.1469	1045.1460	0.9
[c57+Cu(II)]^5+^	1170.6114	1170.6118	−0.3
[c61+Cu(I)]^5+^	1268.0690	1268.0696	−0.4
[c68+Cu(II)]^7+^	999.5258	999.5248	1.1
[c75+Cu(II)]^7+^	1089.1486	1089.1479	0.6
[c75]^7+^	1080.4466	1080.4454	1.1
[c95+Cu(II)]^9+^	1050.5581	1050.5583	−0.2
[c96+Cu(II)]^9+^	1064.7909	1064.7905	0.4
[c139+Cu(I)]^11+^	1313.8364	1313.8377	−1.0
[z4+H]^+^	429.1748	429.1749	−0.3
[z46+H]^3+^	1723.0915	1723.0913	0.1
[z45+H]^3+^	1690.0687	1690.0696	−0.5
[z57+H]^4+^	1518.4317	1518.4329	−0.8
[z65+H]^5+^	1380.2378	1380.2380	−0.2
[z102+H]^8+^	1323.1481	1323.1489	−0.6
[z101+H]^8+^	1302.7652	1302.7661	−0.8
[z113]^9+^	1296.5308	1296.5318	−0.8
[z112+H]^9+^	1282.3046	1282.3062	−1.2
[z102+H]^9+^	1176.2435	1176.2431	0.4
[z140+Cu(I)]^10+^	1450.6188	1450.6190	−0.1
[z140+Cu(I)]^11+^	1318.8360	1318.8374	−1.1
[z140+Cu(I)]^12+^	1209.0169	1209.0177	−0.7
Average			0.33
Standard Dev			0.98
